# New Factors Enhancing the Reactivity of Cysteines in Molten Globule-Like Structures

**DOI:** 10.3390/ijms21186949

**Published:** 2020-09-22

**Authors:** Giorgia Gambardella, Giada Cattani, Alessio Bocedi, Giorgio Ricci

**Affiliations:** Department of Chemical Sciences and Technologies, University of Rome “Tor Vergata”, 00133 Rome, Italy; giorgia.gambardella@gmail.com (G.G.); giada.cattani@gmail.com (G.C.); bcdlss01@uniroma2.it (A.B.)

**Keywords:** oxidative folding, molten globule, cysteine reactivity, chemical kinetics, hydrophobic interaction, transient complex, bovine serum albumin, lysozyme, ribonuclease, chymotrypsinogen

## Abstract

Protein cysteines often play crucial functional and structural roles, so they are emerging targets to design covalent thiol ligands that are able to modulate enzyme or protein functions. Some of these residues, especially those involved in enzyme mechanisms—including nucleophilic and reductive catalysis and thiol-disulfide exchange—display unusual hyper-reactivity; such a property is expected to result from a low p*K*_a_ and from a great accessibility to a given reagent. New findings and previous evidence clearly indicate that p*K*_a_ perturbations can only produce two–four-times increased reactivity at physiological pH values, far from the hundred and even thousand-times kinetic enhancements observed for some protein cysteines. The data from the molten globule-like structures of ribonuclease, lysozyme, bovine serum albumin and chymotrypsinogen identified new speeding agents, i.e., hydrophobic/electrostatic interactions and productive complex formations involving the protein and thiol reagent, which were able to confer exceptional reactivity to structural cysteines which were only intended to form disulfides. This study, for the first time, evaluates quantitatively the different contributions of p*K*_a_ and other factors to the overall reactivity. These findings may help to clarify the mechanisms that allow a rapid disulfide formation during the oxidative folding of many proteins.

## 1. Introduction

Protein cysteines are involved in many critical structural and functional roles that can be performed thanks to the peculiar properties of their sulfhydryl group, which, in its deprotonated form, becomes an active nucleophile. Due to the physiological importance of this residue, it represents an attractive and emerging target for the development and design of covalent ligands, which are able to modulate the function of specific proteins and enzymes. Therefore, it is of paramount importance that a precise knowledge of the factors that influence the reactivity of these residues is obtained [1,2]. It is a common opinion (universally accepted) that this is mainly controlled by their p*K*_a_ and by the accessibility of a given reagent [1,3,4,5,6,7]. In fact, the sulfhydryl group of cysteines is almost inert in its protonated form (except in free-radical reactions), while the thiolate form is the true reactive form. A relevant number of cysteines with functional roles in catalysis have been found to react a hundred- and even thousand-times faster than a free cysteine, but it is only rarely that a quantitative and reasoned analysis of the contribution of a low p*K*_a_ to these unusual reactivities has been made. This study, based on novel experiments and considering old findings, shows that p*K*_a_ is not the main determinant, given that, at physiological pH values, the highest increment of the reactivity due to p*K*_a_ variations cannot exceed two–four-times. Thus, other important factors will be considered in order to discover that, in a few proteins, they assume an almost exclusive prevalence in modulating the reactivity of these residues. Moreover, we demonstrate that hyper-reactivity is not an exclusive feature of functional cysteines and even structural cysteines may have extraordinary reactivity toward many thiol reagents or natural disulfides, which are possibly finalized to a correct and rapid formation of native disulfide bridges during the nascent phase.

## 2. Results

### 2.1. Poor Enhancement of the Cysteine Reactivity Is Due to pK_a_ Perturbations

An extended study, which examined the p*K*_a_ values of cysteines in many polypeptides, determined 9.1 to be a likely p*K*_a_ value for an unperturbed residue [8]. In fact, this value is close to that (8.9–9) which was found for reduced glutathione [8], which contains a cysteine residue in which its carboxylic and amino groups are not free, but are rather engaged in covalent links with glycine and glutamic acid. At the physiological pH value of 7.4, an unperturbed protein cysteine should be present, like active thiolate, in a small but not negligible fraction (α) evaluated as 2% (see Equations (1)–(4)). Thus, the forced deprotonation of a protein cysteine due to a low p*K*_a_, possibly caused by near-positively charged residues, cannot increase its reactivity more than 50-times (see Section 4 for details).

However, the nucleophilicity of the thiolate decreases by lowering its p*K*_a_. At pH 7.4, for a reaction between alkylating compounds and various thiols with different p*K*_a_, a linear decrease should be observed by plotting the logarithm of the second order kinetic constant of the thiolate (*k*_RS_^−^) onto the corresponding p*K*_a_ of the thiols, according to Equation (5).

**Figure 1:**
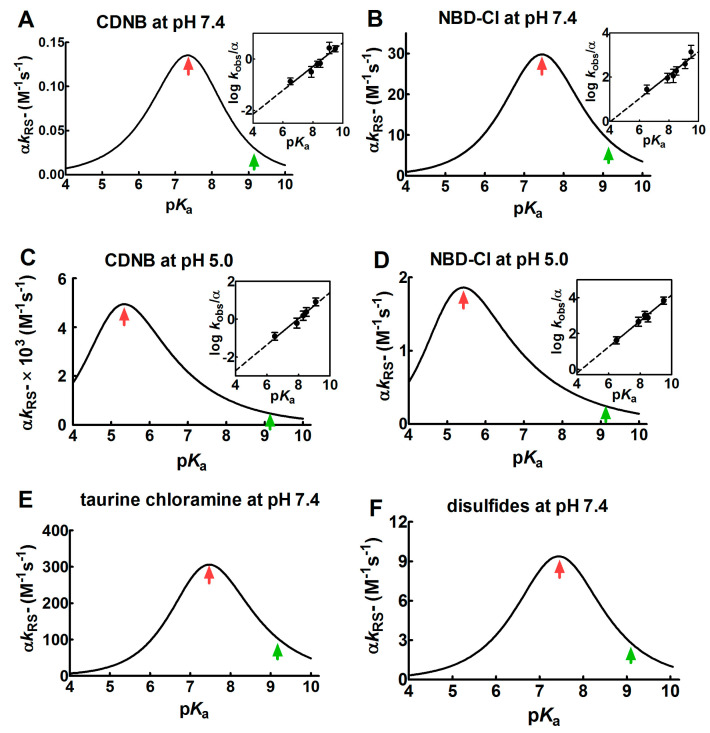
Dependence of kinetic constants on p*K*_a_ for the reaction of thiols with different reagents. (**A**–**D**) Dependence of the observed second order kinetic constants (α *k*_RS_^−^) on p*K*_a_ at pH = 7.4 and pH = 5.0 for the reaction of 1-chloro-2,4-dinitrobenzene (CDNB) and 4-chloro-7-nitrobenzofurazan (NBD-Cl) with thiols with different p*K*_a_ (see Section 4 for details). Continuous lines of bell-shaped curves coming from Equation (6) are obtained from the best-fit of the linear regressions, as shown in the corresponding insets. (**E**,**F**) Dependence of the second order kinetic constants (α *k*_RS_^−^) on p*K*_a_ for the reaction of several thiols with different p*K*_a_ with taurine chloramine and with different disulfides. The data were obtained from previous studies [9,10]. The experimental points of the linear regressions (insets) are the Mean ± S.D. of the three independent experiments. The red arrows mark the maximum values of the bell-shaped graphs. The p*K*_a_ of the unperturbed protein cysteine is labelled with the green arrows.

In our case, this linear relation was confirmed by observing the dependence of log *k*_RS_^−^ on p*K*_a_ in the reaction of different thiols (with different p*K*_a_) with 4-chloro-7-nitrobenzofurazan (NBD-Cl) and with 1-chloro-2,4-dinitrobenzene (CDNB): two well-known alkylating reagents (insets in Figure 1A,B at pH = 7.4 and Figure 1C,D at pH 5.0).

The values of the Brønsted coefficient (*β*_nuc_) and C (a constant applicable to a specific reaction involving various thiols and alkylating reagents) were 0.46 ± 0.07 (*β*_nuc_) and −3.99 ± 0.56 (C) for CDNB and 0.53 ± 0.08 (*β*_nuc_) and −2.13 ± 0.67 (C) for NBD-Cl at pH = 7.4. These values were inserted into the Equation (6), obtaining a bell-shaped behavior with a maximum at p*K*_a_ ≈ 7.4 (Figure 1A,B). This plot also indicates that an unperturbed protein cysteine with p*K*_a_ = 9.1 cannot increase its reactivity more than three–four-times by lowering its p*K*_a_ from 9.1 to 7.4. A further decrease of p*K*_a_ below 7.4 results in a relevant decrease of reactivity. If these reactions are performed at pH = 5.0 (*β*_nuc_ = 0.69 ± 0.07 and *C* = −5.48 ± 0.57 for CDNB, *β*_nuc_ = 0.72 ± 0.06 and *C* = −3.10 ± 0.46 for NBD-Cl), a similar bell-shaped dependence is also observed, but the maximum of the reactivity was shifted to p*K*_a_ ≈ 5.4 (Figure 1C,D). These results parallel those obtained by Ferrer-Sueta et al. for the reaction of the different thiols involved in taurine chloramine reduction at pH = 7.4 (Figure 1E) [9].

A similar variation of the nucleophilicity of the sulfhydryl group was also observed during the reaction of a few disulfides with free thiols or cysteine containing peptides showing different p*K*_a_. Even in that case, a linear decrease was found by plotting the logarithm of the second order kinetic constant of the thiolate onto the corresponding p*K*_a_ of the thiols [9,10,11,12]. From these data, a bell-shaped graph was derived (Figure 1F) as described in Section 4. That study reveals that an unperturbed protein cysteine with a p*K*_a_ = 9.1 cannot increase its reactivity toward disulfides more than three-times at pH = 7.4.

Overall, our experimental data, together with preceding results, indicate that protein cysteines showing p*K*_a_ ≤ 5.0 are almost unreactive toward various thiol reagents at physiological pH values.

### 2.2. Hydrophobic Interactions

**Figure 2:**
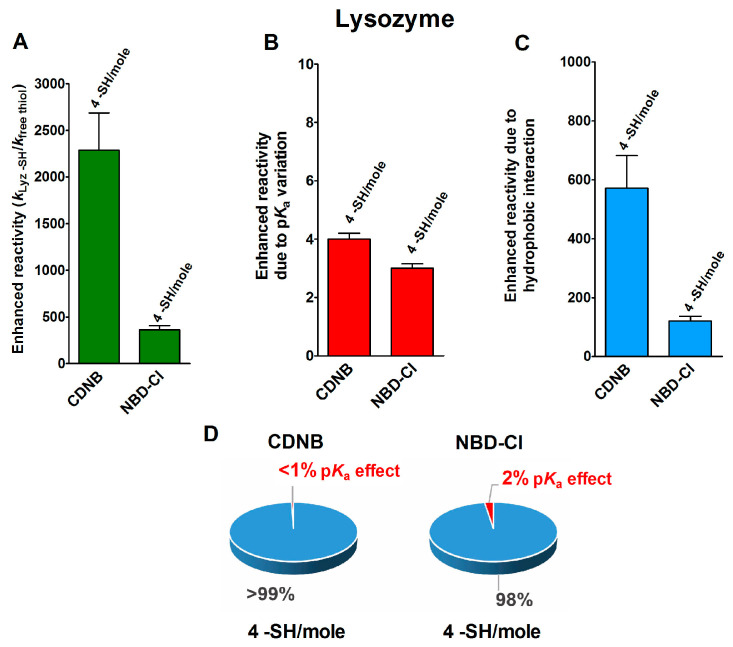
p*K*_a_ and hydrophobic effects on the observed hyper-reactivity in a reduced lysozyme. (**A**) Experimental kinetic data for the reaction of rLyz with CDNB and NBD-Cl at pH = 7.4, normalized to those of an unperturbed protein cysteine (enhanced reactivity) [13]. (**B**) Enhanced reactivity due to the experimental lower p*K*_a_ for four cysteines (p*K*_a_ = 7.1). (**C**) Enhanced reactivity due to hydrophobic interaction. (**D**) The circle diagrams show the percent contribution of the two factors: the hydrophobic effect (blue) and the p*K*_a_ effect (red). The 100% represents the sum of the values reported in panels (**B**,**C**). The error bars in panels (**A**–**C**) are derived from the propagation of uncertainties (see Section 4).

In an interesting experimental set, performed on the reduced forms of a few disulfide-containing proteins and enzymes taken in their corresponding molten globule-like conformations, an extraordinary enhancement of the reaction rates of a few cysteines in their reaction with two thiol reagents with hydrophobic characters, i.e., CDNB and NBD-Cl (Table 1), was observed. As reported in Figure 2A, four cysteines in the reduced lysozyme (rLyz) which also display a low p*K*_a_ (7.1) react with a thousand and a hundred-times higher reactivity with CDNB and NBD-Cl, respectively [13]. After removing the positive contribution of a lowered p*K*_a_ (Figure 2B) (about three-times), 572 and 121 are the enhanced reactivities normalized to the one of an unperturbed protein cysteine (Figure 2C and Table 1). This extra-reactivity is probably due to hydrophobic interactions, as will be confirmed below.

The circle diagrams of Figure 2D compare the percent values of reactivity due to p*K*_a_ variation and the hypothesized hydrophobic effects for both of these reagents.

**Figure 3:**
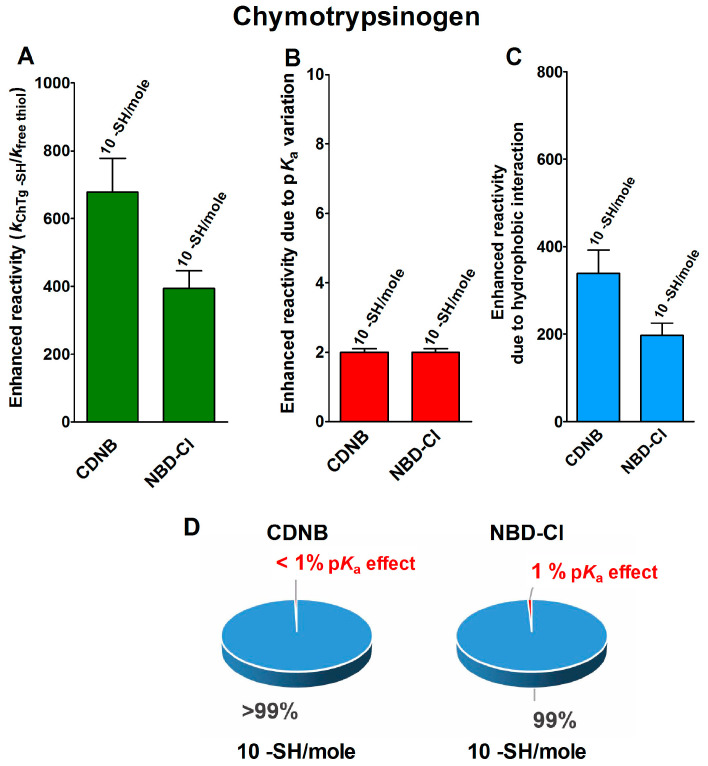
p*K*_a_ and hydrophobic effects on the observed hyper-reactivity in the reduced chymotrypsinogen. (**A**) Experimental kinetic data for the reaction of rChTg with CDNB and NBD-Cl at pH = 5.0, normalized to those of an unperturbed protein cysteine (enhanced reactivity) [16]. (**B**) Enhanced reactivity due to the experimental lower p*K*_a_ for 10 cysteines (p*K*_a_ = 8.1). (**C**) Enhanced reactivity due to hydrophobic interaction. (**D**) The circle diagrams show the percent contribution of the two factors: the hydrophobic effect (blue) and the p*K*_a_ effect (red). The 100% represents the sum of the values reported in panels (**B**,**C**). The error bars in panels (**A**–**C**) are derived from the propagation of uncertainties (see Section 4).

The reduced form of chymotrypsinogen (rChTg) also displays a very similar enhanced reactivity that involves all the ten cysteine residues of this enzyme showing a lowered p*K*_a_ of 8.1 (Figure 3A–C) [16]. For this protein, the experiments were performed at pH = 5.0 due to the irreversible aggregation occurring at higher pH values [16]. The net contribution of the hypothesized hydrophobic interaction may be evaluated as 339- and 197-times enhanced reactivity for CDNB and NBD-Cl, respectively, while that which was due to the lowered p*K*_a_ of these cysteines is only two-times (Table 1).

The circle diagrams allow a percentage comparison of the two effects on the total reactivity of these cysteines with these two reagents (Figure 3D).

**Figure 4:**
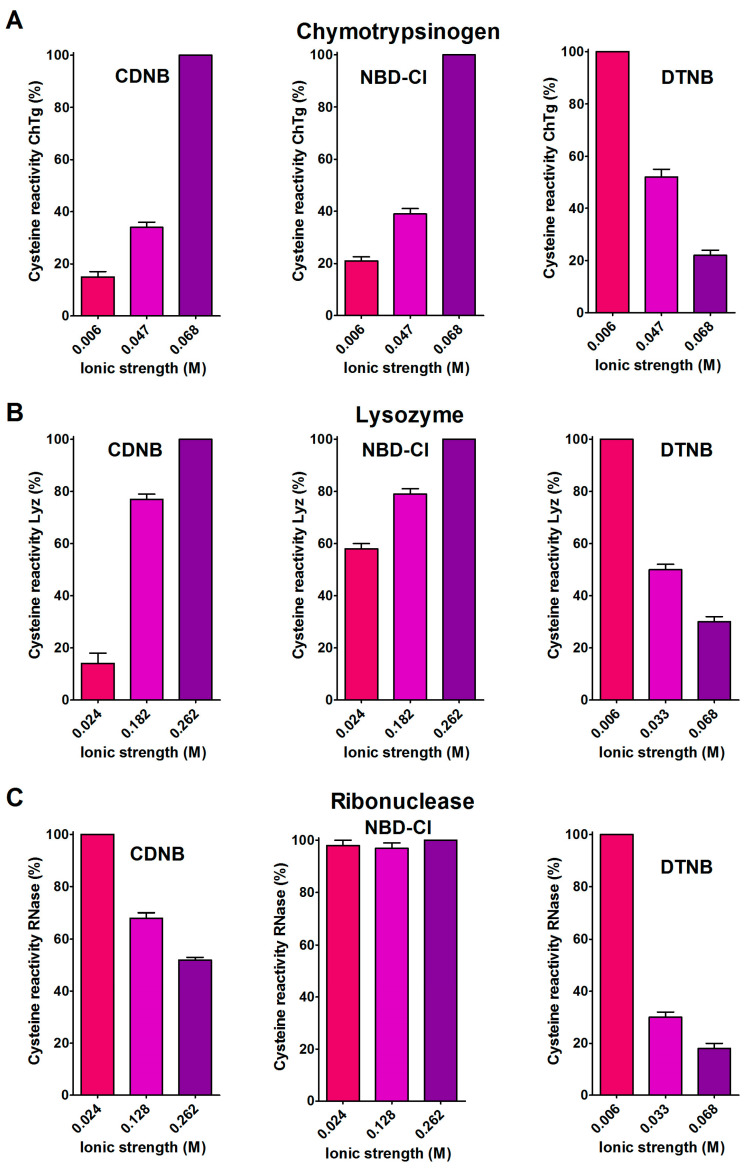
Effect of ionic strength variation on the reactivity of the reduced chymotrypsinogen, lysozyme and ribonuclease. (**A**) The change of the hyper-reactivity due to ionic strength variation was assayed for rChTg’s reaction with CDNB, NBD-Cl and DTNB at pH = 5.0, as reported in Section 4. (**B**) A similar experiment was performed with rLyz for the reaction with CDNB and NBD-Cl at pH = 7.4 and DTNB at pH = 5.0. (**C**) The change of the hyper-reactivity due to ionic strength variation was assayed for rRNase’s reaction with CDNB and NBD-Cl at pH = 7.4 and DTNB at pH = 5.0. The details are reported in Section 4. The data are the Mean ± S.D. of three independent experiments.

A support for the presence of hydrophobic interactions as the cause of the hyper-reactivities found in rChTg and rLyz toward CDNB and NBD-Cl was given by the relevant decrease of the reactivity observed by lowering the ionic strength (Figure 4A,B), as was theoretically predicted [18].

A different and opposite behavior was observed for the reduced ribonuclease (rRNase) in its reactions with CDNB and NBD-Cl at different ionic strengths (Figure 4C). This evidence and the modest hyper-reactivity of this reduced protein toward these reagents, indicates the absence of a hydrophobic interaction which is able to speed up these reactions.

**Figure 5:**
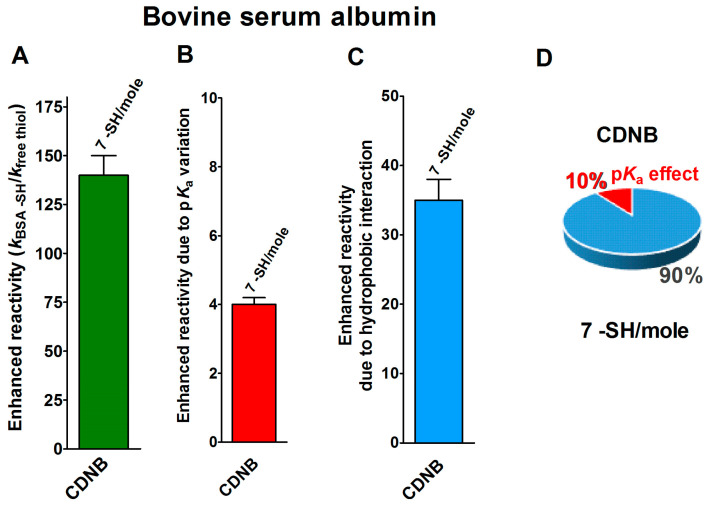
p*K*_a_ and hydrophobic effects on the observed hyper-reactivity in the reduced bovine serum albumin. (**A**) Experimental kinetic data for the reaction of rBSA with CDNB at pH = 7.4, normalized to those of an unperturbed protein cysteine (enhanced reactivity) [14]. (**B**) Enhanced reactivity due to the experimental lower p*K*_a_ for the seven most reactive cysteines (average p*K*_a_ = 7.8). (**C**) Enhanced reactivity due to hydrophobic interaction. (**D**) The circle diagram shows the percent contribution of the two factors: the hydrophobic effect (blue) and the p*K*_a_ effect (red). The 100% represents the sum of the values reported in panels (**B**,**C**). The error bars in panels (**A**–**C**) are derived from the propagation of uncertainties (see Section 4).

Finally, seven cysteines in the reduced form of bovine serum albumin (rBSA) also display 140-times enhanced reactivity toward CDNB, when compared to that of an unperturbed protein cysteine (Figure 5A) [14]. The expected maximum implementation, calculated on the basis of the p*K*_a_ variations (p*K*_a_ = 7.8 for 7 Cys), is about four-times (Figure 5B). Thus, 35-times is the enhanced reactivity due to hydrophobic interactions (Figure 5C and Table 1).

The circle diagram visualizes the percent contributions of the hydrophobic and p*K*_a_ effects (Figure 5D).

### 2.3. The Peculiar Interaction with 5,5′-Dithiobis-(2-Nitrobenzoic Acid)

The 5,5′-dithiobis-(2-nitrobenzoic acid) (DTNB), a well known thiol reagent, displays both polar and non-polar characteristics in the same molecule (two aromatic rings and two ionized carboxylate functions). This particular nature is the origin of its interesting interaction with all of the tested reduced proteins in their molten globule-like structures. As reported in Table 1, a few cysteines of rLyz (7 Cys), rChTg (10 Cys) and rBSA (7 Cys) are hyper-reactive toward this reagent, with a hundred-times enhanced reactivity. The different numbers of hyper-reactive cysteines and the different extents of their increased reactivity compared to those seen with CDNB and NBD-Cl is probably due to the presence of the charged carboxylate. The influence of ionic interactions in the protein cysteine reactivity has been described in the past [5,19] and it appears to be crucial in the case of the rRNase. This protein does not display any hyper-reactivity toward CDNB and NBD-Cl, but almost all of its eight cysteines react with DTNB with 125-times increased reactivity. This phenomenon is likely due only to the electrostatic positive interactions of this reagent with charged residues surrounding all the protein cysteines. The effect of the ionic strength on this hyper-reactivity—exactly the opposite of that shown with CDNB and NBD-Cl (Figure 4C)—confirms a prevalent electrostatic effect. A similar conclusion can be drawn for rChTg and rLyz (Figure 4A,B).

### 2.4. CD Analyses and ANS Fluorescence Fulfill Further Insights

The circular dichroism (CD) spectra for albumin, lysozyme, ribonuclease and chymotrypsinogen, both in their native and in their reduced molten globule-like status, have been reported in previous studies [13,14,15,16]. Now, a more detailed analysis, performed using the BeStSel program [20], can give us further insights about the molten globule-like structures. As shown in Table 2, the conversion of the native oxidized form of albumin, chymotrypsinogen and lysozyme into a reduced molten globule-like structure causes a loss of the alpha helix and an accompanying increase in the beta sheet content. This evidence only suggests some changes of the secondary structures, but nothing about an increased exposition of hydrophobic residues which are able to promote a productive binding of CDNB and NBD-Cl. The considerable fluorescence emission of 8-anilinonaphthalene-1-sulfonic acid (ANS) in the presence of the reduced protein, compared to that observed with the oxidized protein (Table 3), represents a consistent proof that the semi-flexible nature of the molten globule status permits some internal non-polar residues to become exposed to the solvent, making the surface more hydrophobic [21]. Thus, this conformation can bind non-polar molecules much more strongly than the native one, thereby favoring the observed enhanced reactivity toward CDNB and NBD-Cl. Conversely, the rRNase displays a secondary structure similar to the oxidized form and only a negligible binding to ANS. These finding could explain the absence of hyper-reactivity toward CDNB and NBD-Cl (Table 1).

### 2.5. Productive Transient Complex

Beside hydrophobic and electrostatic interactions, other factors may be present in the reduced forms of the proteins, which are able to greatly increase the cysteine reactivity. By examining the reaction of the reduced molten globule-like structures of the four tested proteins with oxidized glutathione (GSSG), we found that a single specific cysteine in each protein displays thousands-times higher reactivity than an unperturbed protein cysteine (Table 1). This phenomenon has been observed in rBSA, rLyz, rRNase and rChTg, for Cys75, Cys94, Cys95 and Cys1, respectively [13,14,15,16]. This hyper-reactivity is specific for GSSG, because a normal reactivity has been found toward other natural disulfides like cystine and cystamine (see Table 1).

**Figure 6:**
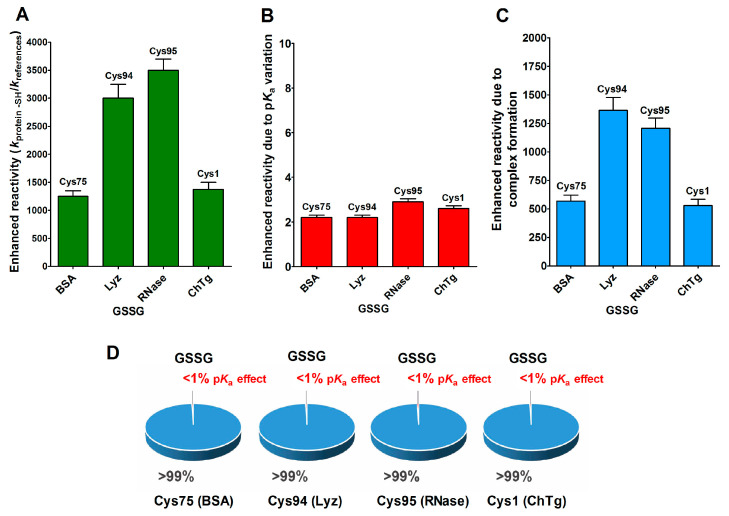
p*K*_a_ and hydrophobic effects on the observed hyper-reactivity toward GSSG in different proteins. (**A**) Experimental kinetic data for the reaction of Cys75 for rBSA, Cys94 for rLyz, Cys95 for rRNase and Cys1 for rChTg with GSSG normalized to those of an unperturbed protein cysteine (enhanced reactivity) [13,14,15,16]. (**B**) Enhanced reactivity due to the experimental lower p*K*_a_ of the protein cysteines of panel (**A**) (p*K*_a_ values: 6.6 for Cys75, 6.6 for Cys94, 7.9 for Cys95 and 8.1 for Cys1) [13,14,15,16]. (**C**) Enhanced reactivity due to a transient complex. (**D**) Circle diagrams show the percent contribution of the two factors: transient complex protein-GSSG (blue) and p*K*_a_ effect (red). The 100% represents the sum of the values reported in panel (**B**,**C**). The error bars in panels (**A**–**C**) derived from the propagation of uncertainties (see Section 4).

This phenomenon is mainly due to a productive transient protein–GSSG complex, as proven by fluorescence and kinetic experiments which also defined the dissociation constants of this interaction (*K*_D_ = 0.3 mM for rLyz, *K*_D_ = 0.12 mM for rRNase and *K*_D_ = 1.5 mM for rChTg) [13,14,15,16]. In other words, a specific region of the molten globule-like conformations of these proteins resembled an enzyme active site which was able to bind productively with GSSG in order to react with a selected cysteine. In Figure 6A–C, we report the reactivity toward GSSG of a single cysteine of these four enzymes, compared to that of an unperturbed protein cysteine (p*K*_a_ = 9.1) together with the enhanced reactivity subtracted by the contribution of a lowered p*K*_a_. In particular, these were p*K*_a_ = 6.6 for Cys75 (rBSA) and Cys94 (rLyz), p*K*_a_ 7.9 = for Cys95 (rRNase) and p*K*_a_ = 8.1 for Cys1 (rChTg) (Table 1).

The circle diagrams (Figure 6D) allow a percent comparison of the effects due to a transient complex formation and p*K*_a_ on the total reactivity of these cysteines with GSSG.

## 3. Discussion

All of the above data lead to the conclusion that p*K*_a_ perturbations represent only a very small contribution for the enhancement of the reactivity in many types of reaction involving a protein cysteine [22]. Previous data [9,10,11,12] gave this indication for thiol–disulfide reactions and for the interaction of thiols with peroxide, but now we have extended the analysis to the reactions involving alkylating compounds. For all of these types of reactions, only three–four-times increased reactivity can be achieved; these are very modest implementations when compared to those found for many functional cysteines exhibiting thousand-times increased reactivity.

Paradoxically, forced deprotonations leading p*K*_a_ < 7.0 provide significant decreases in reactivity. Many studies have often referred to this parameter to justify unusual kinetic properties and this conclusion can be revised. In this paper, we have shown an evident extraordinary effect due to a hydrophobic interaction between two thiol reagents (CDNB, NBD-Cl) and a few structural cysteines devoted to the formation of disulfides in the native proteins (i.e., bovine serum albumin, lysozyme and chymotrypsinogen). It appears that the unknown structures of the molten globule-like conformations of these proteins expose most of their cysteines to these reagents with a concomitant nearness of hydrophobic amino acids, which in the native conformations are normally masked and confined inside the polypeptide radius. The relevant increase of beta sheets in the molten globule-like proteins (Table 2) and the increase of ANS fluorescence in the presence of rChTg and rLyz (Table 3) are signals of the widespread exposed hydrophobicity of these structures, favoring the interaction with CDNB and NBD-Cl. The particular effect of the ionic strength on the reactivity of these cysteines toward these reagents is a further proof of the presence of hydrophobic interactions as the cause of the observed hyper-reactivity. A second accelerating factor must be mentioned, i.e., the electrostatic interaction, which seems to be the origin of the hyper-rectivity toward DTNB observed in rRNase, rLyz and rChTg.

As a third and more evident kinetic-enhancing factor, we discovered the specific interaction of GSSG with only one cysteine residue of each of these proteins. Here, the increased reactivity is evaluated in terms of thousand-times with a stringent specificity: no other small natural disulfides, like cystine and cystamine, show similar reactivities [13,14,15,16]. This hyper-reactivity toward GSSG could reveal a primordial event inside the oxidative folding of many disulfide containing proteins, given that GSSG is present at millimolar concentrations in the endoplasmic reticulum [23]. This should indicate that the glutathionylation of these cysteines could represent a useful “incipit” of their oxidative folding avoiding protein aggregation, as demonstrated for lysozyme [13], or to promote a hierarchical disulfide bond formation, as found in albumin [14].

**Figure 7:**
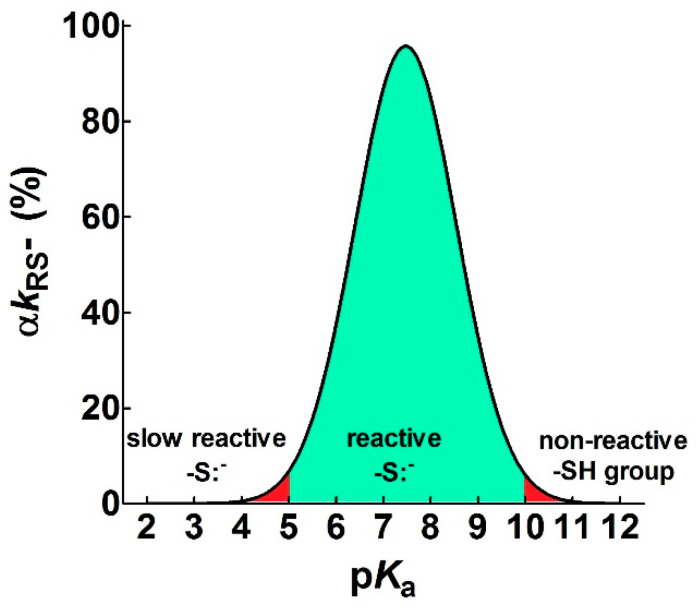
Theoretical curve defining the range of useful p*K*_a_ (α*k*_RS_^−^ ≥ 5%) for a reacting thiolate group. The figure shows the p*K*_a_ range that allows a sufficient rate of reaction of a protein cysteine with different reagents (green area) at pH = 7.4. Values with α*k*_RS_^−^ < 5% are reported in red. The continuous line represents an average of the experimental curves reported in Figure 1.

In conclusion:

- p*K*_a_ is not the main determinant in the enhancement of the reactivity of protein cysteines toward various reagents. Conversely, a very low p*K*_a_, as well as a very high p*K*_a_, may render unreactive these residues (see Figure 1 and Figure 7). What is the utility of some functional cysteine showing very low p*K*_a_, such as selected residues in DsbA (p*K*_a_ = 3.5), DsbC (p*K*_a_ = 4.1) and Grx1 (p*K*_a_ = 3.5) [24]? One reasonable explanation is that this property accelerates the reaction of the oxidized form of these enzymes with the thiol substrates stabilizing the products [24]. Another possibility is that a very low p*K*_a_ that makes the thiolate less reactive and may preserve it against some unproper modifications. This may be the case for GSTP1-1, where the thiolate of Cys47 (p*K*_a_ = 3.5) is bound to Lys54 in an ion-pair, which is important for the enzyme mechanism and a correct binding of the substrate [25].
- Cysteine hyper-reactivity is not an exclusive property of functional cysteines involved in catalysis and even structural cysteines devoted to the formation of disulfides may display hundred- or thousand-times increased reactivity toward GSSG and various thiol reagents.
- Hydrophobic interactions are the main determinant factors triggering hyper-reactivity toward CDNB and NBD-Cl for rBSA, rChTg and rLyz, while electrostatic interactions are the prominent factors for the reactivity of DTNB toward rRNase, rLyz and rChTg.
- A specific binding site for GSSG is surprisingly present in the reduced molten globule-like conformations of albumin, lysozyme, chymotrypsinogen and ribonuclease. It is the main determinant for the observed hundred- and even thousand-times increased reactivity of one specific cysteine. This phenomenon raises the question of whether a rapid glutathionylation may be the early step of their oxidative pathway.
- Methods for the proteomic identification of cysteines, like the isoTOP-ABPP procedure [1,26], should be used with caution, because they only identify hyper-reactive cysteines toward a specific reagent (i.e., a modified iodacetamide) and this property cannot be referred to an ‘intrinsic reactivity’ because it may be not present in reactions with different thiol reagents. Conversely, some protein cysteines, which are normo-reactive toward the modified iodoacetamide probe, can be hyper-reactive toward some natural intracellular compounds. Our data, in fact, likely indicate that one cysteine may have extraordinary hyper-reactivity toward a specific disulfide (GSSG) and normo-reactivity toward other small disulfides, like cystine and cystamine. Conversely, many cysteines which are present in rBSA and rLyz are hyper-reactive toward hydrophobic reagents like CDNB and NBD-Cl, but (except for one residue) are normo-reactive toward GSSG and other small disulfides. In other words, the “intrinsic” reactivity for a protein cysteine is only determined by its p*K*_a_ and by the nucleophilicity of its deprotonated form, but it cannot be increased more than three–four-times, as demonstrated in this paper. An evident hyper-reactivity can only be generated by “extrinsic” factors like the protein environment surrounding the cysteine, which may productively and often selectively bind a specific reagent through hydrophobic or electrostatic interactions.

This selective hyper-reactivity should be of particular interest in the elucidation of the early step of the oxidative folding of these proteins. The hyper-reactivity of protein cysteines appears to be an open puzzle whose pieces, even now, have not been completely identified. This paper may be a useful contribution to this scenario.

## 4. Materials and Methods

### 4.1. Chemicals and Reagents

Lysozyme from chicken egg white (about 100,000 U/mg), α-chymotrypsinogen A from a bovine pancreas, ribonuclease A from a bovine pancreas (Type XII-A, 75–125 Kunitz units/mg protein), l-cysteine, cysteamine, l-glutathione, oxidized glutathione, *N*-acetyl-l-cysteine, cysteinylglycine, l-cysteine ethyl ester, 1-chloro-2,4-dinitrobenzene, 5,5′-dithiobis(2-nitrobenzoic acid), 4-chloro-7-nitrobenzofurazane, dithiotreitol, ethylendiamminotetreaacetic acid, urea, 8-anilinonaphthalene-1-sulfonic acid and all of the other reagents were purchased from Sigma-Aldrich (St. Louis, MO, USA).

### 4.2. Reactions of Thiols with Alkylating Reagents

The reactivity toward CDNB was evaluated spectophotometrically at 340 nm, where the Cys-DNB adduct absorbs (ε = 9.6 mM^−1^ cm^−1^). The reactions were performed at 25 °C by mixing CDNB (1 mM in 0.1 M sodium phosphate buffer, pH = 7.4, or 2 mM in 0.1 M sodium acetate buffer, pH = 5.0) with 0.1 mM of several thiols with different p*K*_a_.

The reactivity of the different thiols (0.1 mM in 0.1 M sodium phosphate buffer, pH = 7.4, or 0.2 mM in 0.1 M sodium acetate buffer, pH = 5.0, 25 °C) toward 0.1 mM NBD-Cl was determined spectrophotometrically at 419 nm, where the Cys-NBD adduct absorbs (ε = 13 mM^−1^ cm^−1^)

The thiols were cysteine ethyl ester (p*K*_a_ = 6.5), cysteinylglycine (p*K*_a_ = 7.9), cysteamine (p*K*_a_ = 8.3), cysteine (p*K*_a_ = 8.5), glutathione (p*K*_a_ = 9.0) and acetylcysteine (p*K*_a_ = 9.5).

### 4.3. Reactivity of rChTg Cysteines toward Alkylating Reagents and DTNB Varying the Ionic Strength

The chymotrypsinogen (ChTg) reduction was performed as previously reported [16]. In brief, 8 mg protein was solubilized in 8 M urea, 1 mM EDTA, 10 mM sodium borate buffer (pH = 8.5) and the ChTg cystine was reduced by adding dithiotreitol (DTT) (ChTg:DTT = 1:21) at 60 °C for 50 min. The DTT excess was removed through a Sephadex G-25 column (1 × 20 cm) equilibrated with 8 M urea, 1 mM EDTA and 10 mM acetate buffer (pH = 5.0). An aliquot of this reduced protein was assayed with DTNB. All of the 10 –SH/mole were titrated at pH = 5.0 in the presence of 0.2 M urea.

The second order kinetic constants for the reactions of the rChTg with CDNB were determined spectrophotometrically, continuously at 340 nm, where the Cys-DNB adduct absorbs (ε = 9.6 mM^−1^ cm^−1^). The concentrations were from 1 to 5 µM rChTg and 0.5 mM CDNB in 0.01 M, 0.07 M or 0.1 M sodium acetate buffer (pH = 5.0).

The reactivity of rChTg (1 µM in sodium acetate buffer 0.1 M or 2 µM in sodium acetate buffer 0.07 M or 0.01 M, pH = 5.0) toward NBD-Cl (50 µM) was evaluated spectrophotometrically, continuously at 419 nm (ε = 13 mM^−1^ cm^−1^), where the Cys-NBD adduct absorbs.

The reactivity of rChTg (0.7 µM) toward DTNB (57 µM) was evaluated spectrophotometrically, continuously at 412 nm (ε = 11.8 mM^−1^ cm^−1^) in 0.1 M, 0.07 M and 0.01 M sodium acetate buffer (pH = 5.0).

The slight turbidity observed in the samples containing 0.1 M buffer was subtracted by each determination.

The ionic strengths calculated for the 0.1 M, 0.07 M and 0.01 M sodium acetate buffer at pH = 5.0 are 0.068 M, 0.047 M and 0.006 M, respectively [27].

### 4.4. Reactivity of rLyz Cysteines toward Alkylating Reagents and DTNB Varying the Ionic Strength

The lysozyme (Lyz) reduction was previously reported in [13]. In brief, Lyz (0.1 mM) was reduced with DTT (10 mM) in 8 M urea, 1 mM EDTA and 10 mM sodium borate buffer, pH = 8.5. After 60 min at 50 °C, the solution was passed through a Sephadex G-25 column (1 × 20 cm) equilibrated with 2 M urea, 1 mM EDTA and 10 mM sodium phosphate buffer (pH = 7.4). An aliquot of this reduced protein was assayed with DTNB. All of the 8 –SH/mole were titrated at pH 8.0 in the presence of 0.2 M urea.

The reactivity of rLyz (1 µM) with CDNB (100 µM) was determined spectrophotometrically in continuous at 340 nm (ε = 9.6 mM^−1^ cm^−1^ for the Cys-DNB adduct) in 0.1 M, 0.07 M or 0.01 M sodium phosphate buffer (pH = 7.4).

The reactivity of rLyz (1 µM) toward NBD-Cl (10 µM) was evaluated spectrophotometrically, continuously at 419 nm (ε = 13 mM^−1^ cm^−1^ for the Cys-NBD adduct) in 0.1 M, 0.07 M or 0.01 M sodium phosphate buffer (pH = 7.4).

The reaction between rLyz (1.24 µM) and DTNB (22.8 µM) was evaluated spectrophotometrically, continuously at 412 nm (ε = 11.8 mM^−1^ cm^−1^) in 0.1 M, 0.05 M and 0.01 M sodium acetate buffer at pH = 5.0.

The slight turbidity observed in samples containing 0.1 M buffer was subtracted by each determination.

The ionic strengths calculated for the 0.1 M, 0.07 M and 0.01 M sodium phosphate buffer at pH 7.4 are 0.262 M, 0.182 M and 0.024 M, respectively [27]. The ionic strength of the 0.1 M, 0.05 M and 0.01 M sodium acetate buffer at pH = 5.0 is 0.068 M, 0.033 M and 0.006 M, respectively [27].

### 4.5. Reactivity of rRNase Cysteines toward Alkylating Reagents and DTNB Varying the Ionic Strength

The ribonuclease (RNase) reduction was performed as previously reported in [15]. In brief, RNase (0.1 mM) was reduced with DTT (10 mM) in 8 M urea, 1 mM EDTA and 10 mM sodium borate buffer (pH = 8.5) at 37 °C for 30 min. The solution was then passed through a Sephadex G-25 column (1 × 20 cm) equilibrated with 2 M urea, 1 mM EDTA and 20 mM sodium phosphate buffer (pH = 7.4). An aliquot of this reduced protein was assayed with DTNB. All of the 8 –SH/mole were titrated at pH = 7.4 in the presence of 0.2 M urea.

The reaction between rRNase (1.29 µM) and CDNB (1 mM) was followed spectrophotometrically, continuously at 340 nm (ε = 9.6 mM^−1^ cm^−1^ for the Cys-DNB adduct) in 0.1 M, 0.05 M or 0.01 M sodium phosphate buffer (pH = 7.4).

The reactivity of rRNase (1.29 µM) toward NBD-Cl (50 µM) was evaluated spectrophotometrically, continuously at 419 nm (ε = 13 mM^−1^ cm^−1^ for the Cys-NBD adduct) in 0.1 M, 0.05 M or 0.01 M sodium phosphate buffer (pH = 7.4).

The reactivity of rRNase (1.27 µM) toward DTNB (20 µM) was determined spectrophotometrically, continuously at 412 nm, where TNBS^−^ absorbs (ε = 11.8 mM^−1^ cm^−1^). The reaction was performed in 0.1 M, 0.05 M or 0.01 M sodium acetate buffer (pH = 5.0).

The ionic strengths calculated for the 0.1 M, 0.05 M and 0.01 M sodium phosphate buffer at pH 7.4 are 0.262 M, 0.128 M and 0.024 M, respectively [27]. The ionic strength of the 0.1 M, 0.05 M and 0.01 M sodium acetate buffer at pH 5.0 is 0.068 M, 0.033 M and 0.006 M, respectively [27].

### 4.6. ANS Fluorescence Assay

ANS was dissolved in 10 mM sodium phosphate buffer (pH = 7.4) at a concentration of 1.9 mM, estimated spectrophotometrically at 450 nm (ε = 4.95 mM^−1^ cm^−1^). The fluorescence measurements of the ANS were performed on a Fluoromax-4 Horiba spectrofluorometer with slits of 2–5 nm, an excitation wavelength of 345 nm and emission spectra of 400–600 nm at 25 °C. The spectra were recorded in 0.2 M urea and 10 mM phosphate buffer at pH = 7.4 (except pH = 5.0 for ChTg) for the oxidized and reduced lysozyme (1.22 µM), ribonuclease (2 µM) and chymotrypsinogen (0.5 µM) after incubation with ANS, with a ratio of protein:ANS = 1:30.

### 4.7. Data Analysis and Graphical Representation

The only active form of a thiol group is its deprotonated form; thus, for an unperturbed protein cysteine with a p*K*_a_ = 9.1 [8]

(1) $$\frac{\left[{\mathrm{CysS}}^{-}\right]\left[H_{3}O^{+}\right]}{\left[\mathrm{CysSH}\right]}=10^{-9.1}$$

Moreover, the fraction of dissociated sulfhydryl is

(2) $$\frac{\left[{\mathrm{CysS}}^{-}\right]}{\left[{\mathrm{CysS}}^{-}\right]+\left[\mathrm{CysSH}\right]}=\alpha$$

Combining these two equations,

(3) $$\alpha =\frac{\frac{\left[{\mathrm{CysS}}^{-}\right]}{\left[\mathrm{CysSH}\right]}}{\frac{\left[{\mathrm{CysS}}^{-}\right]}{\left[\mathrm{CysSH}\right]}+1}=\frac{10^{\mathrm{pH}-pK_{a}}}{1+10^{\mathrm{pH}-pK_{a}}}$$

At pH = 7.4, α is 0.020; thus, a lowered p*K*_a_ of the sulfhydryl group, which makes an almost fully dissociated cysteine (α = 1), cannot cause a kinetic increase higher than 50-times at physiological pH values.

A typical reaction of a protein or free cysteine with alkylating reagents can be schematized as:

CysSH+RX→no reaction

$${\mathrm{CysS}}^{-}+\mathrm{RX}\overset{k_{{\mathrm{RS}}^{-}}}{\to }\mathrm{CysSR}+X^{-}$$

The pH-independent rate constant *k*_RS_^−^ of the sulfhydryl group is obtained by dividing the observed/experimental rate constant (*k*_obs_) at a given pH by the fraction of the dissociated sulfhydryl (α) at the same pH:

(4) kRS−=kobsα=kobs(1+10pKa−pH)

The logarithm of the rate constant is linearly correlated with the thiol p*K*_a_ according to the Brønsted relationship:

(5) $$\log k_{{\mathrm{RS}}^{-}}=\beta_{\mathrm{nuc}}pK_{a}+C$$

where *β*_nuc_ is the Brønsted coefficient and *C* is a constant applicable to a specific reaction involving various thiols and an alkylating reagent. The data of the linear regressions (insets of Figure 1) are expressed as Mean ± Standard Deviation (S.D.). The data were obtained from three independent experiments. 

To obtain the bell-shaped graphs reported in Figure 1, Equations (4) and (5) were combined:

(6) $$\alpha k_{{\mathrm{RS}}^{-}}=k_{\mathrm{obs}}=10^{\beta_{\mathrm{nuc}}pK_{a}+C-\log\left(1+10^{pK_{a}-\mathrm{pH}}\right)}$$

Parameters ($\beta_{\mathrm{nuc}}$ and *C*) derived from the linear correlations between log *k*_RS_^−^ and peptide thiols p*K*_a_ in the thiol-disulfide exchange reactions [10] were inserted into Equation (6), thus obtaining a bell-shaped graph (Figure 1F). A similar procedure was used for the analysis of the taurine chloramine reduction by several small thiols [9], obtaining the bell-shaped graph shown in Figure 1E.

The experimental data from the literature [9,10] were digitalized using GetData Graph Digitizer software (v2.24) (ShareIt, Germany). The propagation of uncertainties was analyzed according to the classical statistical methods [28]. The graphics and the results visualization were obtained using GraphPad Prism software v5.0 (GraphPad Company, La Jolla, CA, USA).

## Figures and Tables

**Table 1:** Enhanced reactivity and analytical data for the reaction of several thiol reagents with the four reduced proteins in their molten globule-like structures.

Thiol Reagent	Thiol	Exp. pH	Reactive Cysteines	p*K*_a_	*k* (M^−1^s^−1^)	Total E.R. ^a^	E.R. Due to p*K*_a_	E.R. Due to Other Factors
CDNB	GSH	7.4		9.0	0.07	1		
	GSH	5.0		9.0	0.00028	1		
	rBSA	7.4	7	7.8 ^b^	9.8 ^b^	140 ^b^	4	35
	rLyz	7.4	4	7.1 ^c^	160 ^c^	2286 ^c^	4	572
	rRNase	7.4	8	7.9 ^d^	0.9 ^d^	13 ^d^	3	4.3
	rChTg	5	10	8.1 ^e^	0.19 ^e^	678 ^e^	2	339
NBD-Cl	GSH	7.4		9.0	8	1		
	GSH	5.0		9.0	0.032	1		
	rLyz	7.4	4	7.1 ^c^	2900 ^c^	362.5 ^c^	3	121
	rRNase	7.4	8	7.9 ^d^	18 ^d^	2.3 ^d^	3	0.8
	rChTg	5.0	10	8.1 ^e^	12.6 ^e^	394 ^e^	2	197
DTNB ^f^	GSH	6.0		9.0	150	1		
	GSH	5.0		9.0	20	1		
	rBSA	6.0	7	7.8 ^b^	30,000 ^b^	200 ^b^	4.3	46
	rLyz	5.0	7	6.6 ^c^	3200 ^c^	160 ^c^	3.3	48
	rRNase	5.0	8	7.9 ^d^	2500 ^d^	125 ^d^	3.5	36
	rChTg	5.0	1	8.1 ^e^	25,000 ^e^	1250 ^e^	3.5	357
	rChTg	5.0	9	8.1 ^e^	2300 ^e^	115 ^e^	3.5	33
GSSG	Cys	7.4		9.1 ^g^	0.2 ^g^	1 ^g^		
	Cys	5.0		9.1 ^g^	0.0008 ^g^	1 ^g^		
	rBSA	7.4	Cys75	6.6 ^b^	250 ^b^	1250 ^b^	2.2	568
	rLyz	7.4	Cys94	6.6 ^c^	600 ^c^	3000 ^c^	2.2	1364
	rRNase	7.4	Cys95	7.9 ^d^	700 ^d^	3500 ^d^	2.9	1207
	rChTg	5.0	Cys1	8.1 ^e^	1.1 ^e^	1375 ^e^	2.6	529
Cystamine	GSH	7.4		9.0	55	1		
	GSH	5.0		9.0	0.22	1		
	rLyz	7.4	4	6.6 ^c^	43 ^c^	0.8 ^c^	2.2	0.4
	rRNase	7.4	3	7.9 ^d^	250 ^d^	4.5 ^d^	2.9	1.5
	rChTg	5.0	5	8.1 ^e^	0.05 ^e^	0.23 ^e^	2.6	0.09
Cystine	GSH	7.4		9.0	12	1		
	GSH	5.0		9.0	0.048	1		
	rLyz	7.4	1	6.6 ^c^	770 ^c^	64 ^c^	2.2	29
	rRNase	7.4	6	7.9 ^d^	53 ^d^	4.4 ^d^	2.9	1.5
	rChTg	5	8	8.1 ^e^	0.56 ^e^	12 ^e^	2.6	4.6

^a^ E.R., enhanced reactivity is the second order kinetic constant for a given reaction, normalized to that of an unperturbed protein cysteine; ^b^ from Ref. [14]; ^c^ from Ref. [13]; ^d^ from Ref. [15]; ^e^ from Ref. [16]; ^f^ E.R. due to p*K*_a_ from Ref. [17]; ^g^ unperturbed protein cysteine from Ref. [8]. Abbreviations: rBSA, reduced bovine serum albumin; CDNB, 1-chloro-2,4-dinitrobenzene; rChTg, reduced chymotrypsinogen; DTNB, 5,5′-dithiobis-(2-nitrobenzoic acid); GSH, reduced glutathione; GSSG, oxidized glutathione; rLyz, reduced lysozyme; NBD-Cl, 4-chloro-7-nitrobenzofurazan; rRNase, reduced ribonuclease.

**Table 2:** Secondary structure analysis of the CD spectra ^a^.

Secondary Structure	Oxidized BSA	Reduced BSA	Oxidized Lyz	Reduced Lyz	Oxidized RNase	Reduced RNase	Oxidized ChTg	Reduced ChTg
Helix	70%	54%	50%	6%	22%	27%	12%	10%
Strand	0%	16%	24%	27%	20%	24%	25%	39%
Turn	9%	2%	11%	14%	15%	12%	14%	11%
Others	21%	28%	15%	53%	43%	37%	49%	40%

^a^ The CD spectra reported in previous studies [13,14,15,16] were analyzed using the BeStSel program. Abbreviations: BSA, bovine serum albumin; ChTg, chymotrypsinogen; Lyz, lysozyme; RNase, ribonuclease.

**Table 3:** ANS fluorescence ^a^.

Proteins	*F*_c_^red^/*F*_c_^ox^
rLyz	4.4 ± 0.1
rRNase	1.0 ± 0.1
rChTg	11.3 ± 0.2

^a^ Values represent the ANS fluorescence after the reaction with reduced proteins, normalized to that of the oxidized proteins. Abbreviations: rChTg, reduced chymotrypsinogen; rLyz, reduced lysozyme; rRNase, reduced ribonuclease.

## Abbreviations

ANS	8-anilinonaphthalene-1-sulfonic acid
BSA	bovine serum albumin
CD	circular dichroism
CDNB	1-chloro-2,4-dinitrobenzene
ChTg	chymotrypsinogen
DTNB	5,5′-dithiobis(2-nitrobenzoic acid)
DTT	dithiotreitol
EDTA	ethylendiamminotetreaacetic acid
GSH	reduced glutathione
GSSG	oxidized glutathione
Lyz	lysozyme
NBD-Cl	4-chloro-7-nitrobenzofurazan
rBSA	reduced bovine serum albumin
rChTg	reduced chymotrypsinogen
rLyz	reduced lysozyme
RNase	ribonuclease
rRNase	reduced ribonuclease
TNBS^−^	5-thio-2-nitrobenzoate
